# Management of blood lipids in post-kidney transplant patients: a systematic review and network meta-analysis

**DOI:** 10.3389/fphar.2024.1440875

**Published:** 2024-10-08

**Authors:** Bohan Luo, Shan Zhong, Xiaoxiao Wang, Pu Guo, Yifu Hou, Wenjia Di

**Affiliations:** ^1^ School of Medicine, University of Electronic Science and Technology of China, Chengdu, China; ^2^ Organ Transplantation Center, Sichuan Provincial People’s Hospital, University of Electronic Science and Technology of China, Chengdu, Sichuan, China

**Keywords:** post-kidney transplantation, lipid-lowering drug therapy, lipid profiles, adverse events, mortality rate, meta-analysis

## Abstract

**Introduction:**

The primary objective of this systematic review was to provide an overview of the efficacy and safety of various lipid-lowering therapies in patients post-kidney transplant (PKT), given the limited existing literature. Considering the restricted number of available studies, this work aimed to summarize the existing evidence regarding the effectiveness of different lipid-lowering treatments in PKT patients. The effects of various lipid-lowering therapeutic regimens on lipid levels were compared, and their safety was assessed, with the heterogeneity of treatment protocols acknowledged.

**Material and Methods:**

Randomized controlled trials investigating different treatment regimens (DTRs) for regulating lipid levels in PKT patients were systematically retrieved from PubMed, Cochrane Library, and Embase, from inception to March 2024. Literature quality was assessed employing the Cochrane risk of bias assessment tool. Data analysis and graphical representation were performed employing *RevMan5.3* and *Stata 20.0*. The surface under the cumulative ranking area (SUCRA) compared the effects of DTRs on lipid profiles, incidence of adverse events, and all-cause mortality in PKT patients.

**Results:**

Fifteen studies were included, comprising 5,768 PKT patients and involving 9 treatment regimens. The results revealed that, for changes in high-density lipoprotein cholesterol (HDL-C), the SUCRA rankings from highest to lowest among PKT patients receiving DTRs were statins + ezetimibe (70%), placebo (61.5%), fibrates (57.2%), statins (44.1%), and fish oil (17.3%). Regarding changes in low-DL-C (LDL-C), the SUCRA rankings from highest to lowest among PKT patients receiving DTRs were statins (68.2%), statins + ezetimibe (67.5%), fish oil (53.4%), fibrates (34.5%), and placebo (26.5%). For the change in total cholesterol (TC) levels, a network meta-analysis (NMA) revealed that among PKT patients receiving DTRs, the SUCRA rankings from highest to lowest for TC change were statins + ezetimibe (97.6%), proprotein convertase subtilisin/kexin type 9 inhibitors (PCSK9 inhibitors) (74.3%), fish oil (64.3%), statins (61.6%), fibrates (47.2%), placebo (31.6%), calcineurin phosphatase inhibitors (11.9%), and immunosuppressants (11.4%). Regarding the change in triglyceride (TG) levels, a NMA showed that among PKT patients receiving DTRs, the SUCRA rankings from highest to lowest for TG change were fibrates (99.9%), statins (68.9%), PCSK9 inhibitors (66.6%), statins + ezetimibe (55.1%), placebo (49.2%), fish oil (45.0%), immunosuppressants (7.8%), and calcineurin phosphatase inhibitors (7.6%). For the occurrence of kidney transplant failure, a NMA revealed that among PKT patients receiving DTRs, the SUCRA rankings from highest to lowest for reducing the incidence of kidney transplant failure were PCSK9 inhibitors (69.0%), calcineurin phosphatase inhibitors (63.0%), statins (61.5%), placebo (55.1%), steroids (51.8%), immunosuppressants (27.1%), and fibrates (22.5%). Regarding all-cause mortality, a NMA showed that among PKT patients receiving DTRs, the SUCRA rankings from highest to lowest for reducing all-cause mortality were PCSK9 inhibitors (90.5%), statins (55.8%), and placebo (3.7%).

**Conclusion:**

In reducing lipid levels in PKT patients, combination therapy with statins and ezetimibe demonstrated notable advantages and higher effectiveness. PCSK9 inhibitors exhibited greater advantages in reducing adverse events and mortality rates in PKT patients, with higher safety.

## 1 Introduction

Dyslipidemia is one of the more common complications observed in patients following kidney transplantation. It not only directly impacts the long-term survival rate of the transplanted kidney but also increases the risk of cardiovascular diseases and post-transplant complications. Post-transplant dyslipidemia typically includes characteristics such as elevated cholesterol, increased triglycerides, elevated low-density lipoprotein (LDL), and reduced high-density lipoprotein (HDL). These dyslipidemic alterations are often closely associated with immunosuppressive therapy and the inflammatory state related to chronic kidney disease. Immunosuppressants can directly or indirectly affect lipid metabolism, leading to alterations in blood lipid levels (Williams et al., 2001; Weir et al., 2015).

Common lipid-lowering medications include statins, ezetimibe, proprotein convertase subtilisin/kexin type 9 (PCSK9) inhibitors, niacin, and fibrates. Statins, the most used class of lipid-lowering drugs, reduce cholesterol synthesis by inhibiting 3-hydroxy-3-methylglutaryl coenzyme A reductase. Research by Goldberg et al. (1996) demonstrated that statins significantly lower low-density lipoprotein cholesterol (LDL-C) levels and possess anti-inflammatory properties, which can delay the progression of atherosclerosis. This combined effect ultimately reduces the risk of severe cardiovascular events such as myocardial infarction and stroke. Ezetimibe, a representative drug in the ezetimibe class, primarily lowers blood lipid levels by inhibiting intestinal cholesterol absorption (Vanrenterghem et al., 2011). Research by Pascual et al. (2005a) indicated that the combination of ezetimibe and statins results in a more significant reduction in blood lipids and improves therapeutic outcomes. Proprotein convertase subtilisin/kexin type 9 (PCSK9) inhibitors, a rapidly advancing class of biotechnology products, function by inhibiting PCSK9 activity, which significantly increases the expression levels of low-density lipoprotein receptors (LDLR) on hepatocyte surfaces, thereby markedly reducing LDL-C. Despite their high cost, these drugs have demonstrated exceptional lipid-lowering efficacy in patients at extremely high or very high risk, and are often used in combination with statins (Rodriguez et al., 1997; Tedesco-Silva et al., 2019). Niacin and its derivatives, collectively referred to as niacin-based drugs, modulate lipid metabolism through multiple mechanisms and are commonly used in conjunction with other lipid-lowering agents (Stallone et al., 2005). Niacin-based drugs not only reduce LDL-C and triglycerides (TG) but also increase high-density lipoprotein cholesterol (HDL-C). Zhu et al. (2023) reported a high incidence of adverse effects associated with niacin-based medications, which may limit their clinical application. However, their efficacy in specific patient populations remains significant. Fibrates are primarily used to treat hypertriglyceridemia and low HDL-C levels, effectively reducing cholesterol levels and increasing HDL-C (Chen et al., 2008). Commonly used fibrates include fenofibrate and bezafibrate. These medications function by activating peroxisome proliferator-activated receptor alpha (PPARα), which regulates lipoprotein metabolism and has important implications for improving cardiovascular outcomes (Baños et al., 2008). In post-kidney transplant (PKT) patients, drug management is particularly complex and requires careful selection of appropriate lipid-lowering agents based on individual patient characteristics and specific conditions to ensure drug safety. For instance, in patients with post-transplant hypercholesterolemia, statin therapy necessitates close monitoring of liver function and muscle enzyme levels to prevent adverse drug reactions. Ezetimibe and PCSK9 inhibitors also show promising potential in this patient population, but adjustments based on individual tolerability are necessary (Coco et al., 2012). Currently, research on lipid management strategies and pharmacotherapy in PKT patients is relatively limited. Existing studies primarily focus on the application and efficacy of various lipid-lowering medications in clinical practice. However, evidence on the specific efficacy, safety, and long-term prognosis of these treatments in post-transplant patients remains insufficient. Therefore, it is crucial to conduct systematic research to explore lipid management strategies in PKT patients, aiming to optimize treatment regimens while reducing cardiovascular risk and post-transplant complications.

This work was to demonstrate the efficacy and safety of different lipid-lowering medications in PKT patients, comparing their effects on dyslipidemia and the long-term impact on renal transplant function and cardiovascular health. Through this research, we aimed to provide more effective drug treatment strategies and clinical guidance for lipid management in PKT patients, further improving patient prognosis and quality of life.

## 2 Methodologies

### 2.1 Criteria for literature inclusion and exclusion

Inclusion criteria: i) Study type: double-blind or single-blind randomized controlled trials (RCTs); ii) Study population: PKT patients; iii) Intervention: experimental groups receiving different treatment regimens (DTRs) with intervention and follow-up durations of more than 1 month, while control groups receive placebos or various treatment medications compared to the experimental group; iv) Outcome measures: changes in lipid-related indicators before and after treatment, adverse reactions, mortality, etc.

Exclusion criteria: i) Study participants with other renal diseases or severe comorbidities that may affect outcome indicators; ii) Literature such as reviews, case reports, and conference abstracts; iii) Literature with unclear or unextractable outcome indicators or outcome data; iv) Literature with unclear intervention measures; v) Literature not in Chinese or English languages.

### 2.2 Literature retrieval strategy

Using computerized searches, relevant literature published from the inception of the databases up to March 2024 was retrieved from databases PubMed, Cochrane Library, Embase, etc. MeSH (Medical Subject Headings) terms were employed in combination with “AND” and “OR” logical operators for the search. The PubMed search strategy was as follows: “Kidney Transplantation”(Mesh) OR (((((((((((Kidney Transplantation[Title/Abstract]) OR (Renal Transplantation[Title/Abstract])) OR (Renal Transplantations[Title/Abstract])) OR (Transplantations, Renal[Title/Abstract])) OR (Transplantation, Renal[Title/Abstract])) OR (Grafting, Kidney[Title/Abstract])) OR (Kidney Grafting[Title/Abstract])) OR (Transplantation, Kidney[Title/Abstract])) OR (Kidney Transplantations[Title/Abstract])) OR (Transplantations, Kidney[Title/Abstract])) OR (Lipid Regulating Agents[Title/Abstract]))) AND (“Lipid Regulating Agents”[Mesh])) OR ((((((Lipid Regulating Agents[Title/Abstract]) OR (Agents, Lipid Regulating[Title/Abstract])) OR (Regulating Agents, Lipid[Title/Abstract])) OR (Lipid Regulating Drugs[Title/Abstract])) OR (Drugs, Lipid Regulating[Title/Abstract])) OR (Regulating Drugs, Lipid[Title/Abstract]))) AND “Randomized Controlled Trial”. For Cochrane Library and Embase, slight adjustments were made to the search keywords.

### 2.3 Data extraction

Two authors independently conducted literature search, screening, and full-text review regarding the above criteria. Relevant data were independently extracted by authors into a pre-designed Excel spreadsheet. Extracted data included publication year, first author name, sample size, study design, intervention measures, and outcome indicators. Outcome indicators comprised lipid indicators (high-density lipoprotein cholesterol (HDL-C), low-DL-C (LDL-C), total cholesterol (TC), and TG), incidence rates of adverse events (including cardiovascular events, kidney transplant failure, acute rejection reactions), and all-cause mortality. Continuous variables were denoted as mean ± standard deviation, while categorical variables as frequencies. In case of discrepancies, consensus was reached through discussion or consultation with a third independent author.

### 2.4 Literature quality evaluation

The two authors utilized the Cochrane risk of bias assessment tool (Martimbianco et al., 2023) to evaluate the quality and risk of bias in the included study literature. The assessment criteria included random sequence generation (selection bias), allocation concealment (selection bias), blinding of participants and personnel (performance bias), blinding of outcome assessment (detection bias), incomplete outcome data (attrition bias), selective reporting (reporting bias), and other biases. Each bias assessment was categorized as “Low risk,” “Unclear risk,” or “High risk.”

### 2.5 Statistical analysis

The included studies were assessed for literature quality employing *RevMan5.3*, and network meta-analysis (NMA) was conducted employing *Stata 20.0*. Binary variables were expressed as odds ratios (OR), while continuous variables as mean differences (MD), with effect sizes evaluated along with their 95% confidence intervals (CI). Network evidence plots were generated, with the size of the nodes representing the sample size of each intervention, and the thickness of the lines indicating the amount of direct evidence between interventions. In the presence of closed loops in the network plot, a loop consistency test or node-splitting methodology was adopted for inconsistency analysis to examine differences between direct and indirect comparison results. If no inconsistency was observed, a consistency model was employed for fitting analysis. In case of inconsistency, an inconsistency model was utilized for fitting analysis, followed by sensitivity analysis to assess result stability. The surface under the cumulative ranking area (SUCRA) curve was utilized to rank the outcome indicators of each intervention, and a “comparison-corrected” funnel plot was generated to evaluate small sample effects and publication bias in included studies.

## 3 Results

### 3.1 Literature retrieval process

**FIGURE 1:**
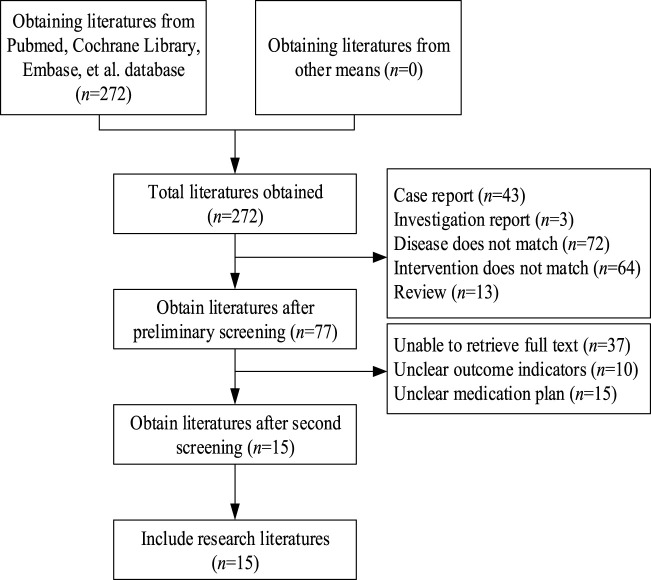
Flowchart of literature selection.

A total of 272 relevant articles were retrieved from PubMed, Cochrane Library, and Embase. After removing duplicates, 243 articles remained. Upon title and abstract screening, 166 articles were excluded due to reasons such as case reports, reviews, interventions not meeting criteria, diseases not meeting study requirements, and other non-compliance issues. Subsequently, 77 articles underwent further screening. Full-text articles were obtained and after thorough reading, 62 articles were excluded due to unclear outcome indicators, undefined treatment regimens, or unavailability of data. Finally, 15 articles (Alotaibi et al., 2024; Castro et al., 1997; Eris, 2005; Flechner et al., 2002; Hausberg et al., 2001; Holdaas et al., 2001; Holdaas et al., 2011; Jardine et al., 2004; Kasiske et al., 2001; Lebranchu et al., 2009; Montagnino et al., 2008; Serón et al., 2008; Sharif et al., 2009; van Hooff et al., 2003; Kohnle et al., 2006) were included. The literature search process is illustrated in Figure 1.

### 3.2 Basic information

Fifteen relevant articles were included, comprising a total of 5,768 patients across 9 treatment modalities. All studies involved renal transplant recipients as the study population. Table 1 presents the basic information of the included studies.

**TABLE 1:** Basic information.

Author	Year	Sample size	Intervention measures		Number of included cases		Outcome indicators
			Intervention group	Control group	Intervention group	Control group	
Alotaibi	2024	197	PCSK9 inhibitors	Statins	98	99	CDEFH
Castro	1997	43	Statins	F	25	18	ABCD
Eris	2005	2,102	Statins	P	1,050	1,052	ABCDF
Flechner	2002	61	CNI	Immunosuppressants (IS)	31	30	EFGH
Hausberg	2001	36	Statins	P	18	18	ABCD
Holdaas	2001	364	Statins	P	182	182	ABCDEH
Holdaas	2011	267	CNI	P	144	123	CDFH
Jardine	2004	2,102	Statins	P	1,050	1,052	ABCD
Kasiske	2001	88	BC	P	36	52	ABCDF
Lebranchu	2009	192	CNI	IS	95	97	BCDF
Montagnino	2008	133	Steroids	P	65	68	F
Sero´n	2008	74	Statins	P	39	35	ABCDFG
Sharif	2009	20	Statins	P	10	10	ABCD
Van Hooff	2003	53	IS + CNI	IS + Statins	25	28	G
Kohnle	2006	36	Statins	Statins + Cholesterol absorption inhibitors (CAI)	18	18	ABCD

Note: A, HDL-C; B, LDL-C; C, TC; D, TG; E, cardiovascular adverse events; F, renal transplant dysfunction; G, acute rejection reaction; H, All-Cause Mortality Rate. (PCSK9 stands for Proprotein Convertase Subtilisin/Kexin Type 9 Inhibitors; Statins refer to Statin Medications; IS, denotes Immunosuppressants; CNI, stands for Calcineurin Inhibitors; BC, refers to Bile Acid Sequestrants; F represents Fibrates; Statins + CAI, means Statins Combined with Ezetimibe; Steroids refer to Corticosteroids; and P stands for Placebo.).

### 3.3 Quality assessment

The bias analysis results of the included literature are presented in Supplementary Figures S1, S2, indicating low risk of bias across the included studies.

### 3.4 Meta-analysis results

#### 3.4.1 NMA results of the impact of DTRs on change in HDL-C levels in PKT patients

**FIGURE 2:**
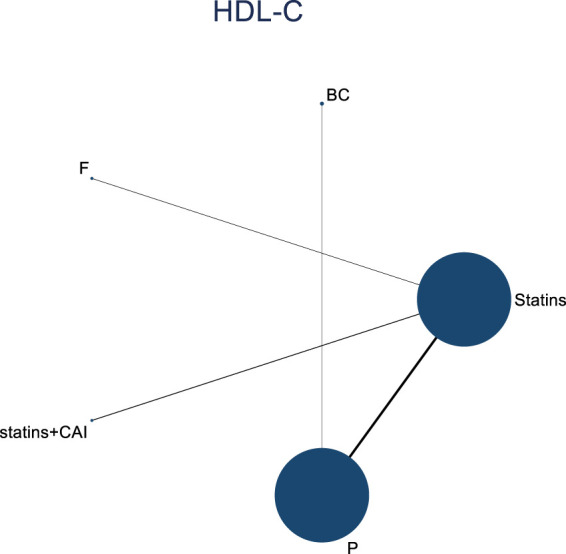
Network evidence relationship diagram illustrating the effect of DTRs on the change in HDL-C levels in PKT patients. (Statins denote Statin Medications; BC stands for Bile Acid Sequestrants; F refers to Fibrates; Statins + CAI means Statins Combined with Ezetimibe; and P represents Placebo).

**FIGURE 3:**
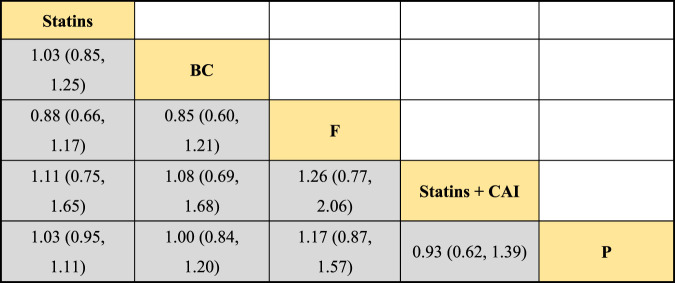
NMA results of the effect of DTRs on change in HDL-C levels in PKT patients. (Statins denote Statin Medications; BC stands for Bile Acid Sequestrants; F refers to Fibrates; Statins + CAI means Statins Combined with Ezetimibe; and P represents Placebo).

**FIGURE 4:**
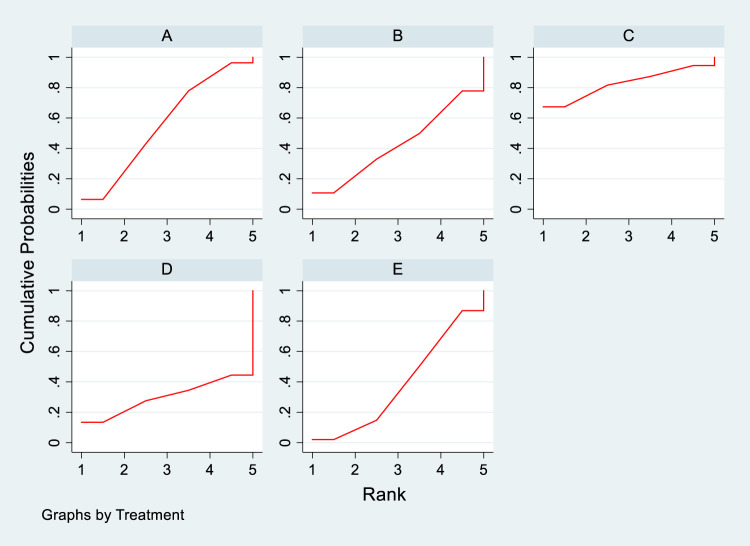
SUCRA plot depicting change in HDL-C levels among PKT patients treated with different therapeutic regimens [**(A)** Statins, **(B)** BC, **(C)** Fibrates (F), **(D)** Statins + CAI, **(E)** Placebo (P)].

In the included literature, 9 studies analyzed the effects of DTRs on HDL-C levels in PKT patients, involving a total of 4,865 patients and comprising 5 intervention measures, including statins, fibrates, fish oil, statins + ezetimibe, and placebo. NMA of HDL-C level changes revealed that the SUCRA ranking from highest to lowest among PKT patients receiving DTRs was as follows: statins + ezetimibe (70%), placebo (61.5%), fibrates (57.2%), statins (44.1%), and fish oil (17.3%) (Supplementary Figure S3; Figures 2–4).

#### 3.4.2 II.NMA results of the influence of DTRs on LDL-C change magnitude in PKT patients

**FIGURE 5:**
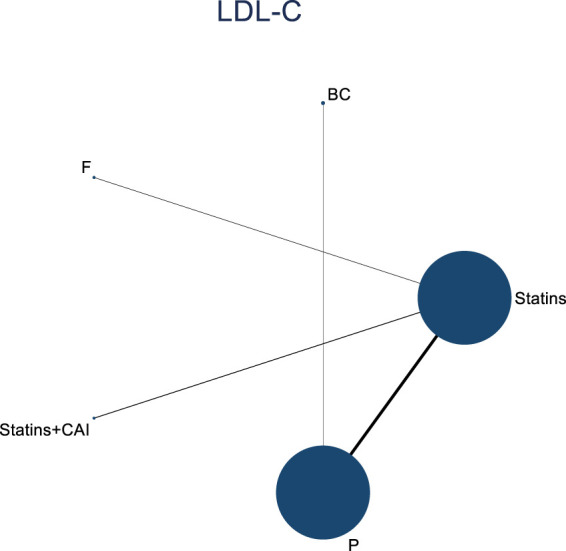
Network evidence diagram illustrating the impact of DTRs on the change in LDL-C levels in PKT patients. (Statins denote Statin Medications; BC stands for Bile Acid Sequestrants; F refers to Fibrates; Statins + CAI means Statins Combined with Ezetimibe; and P represents Placebo).

**FIGURE 6:**
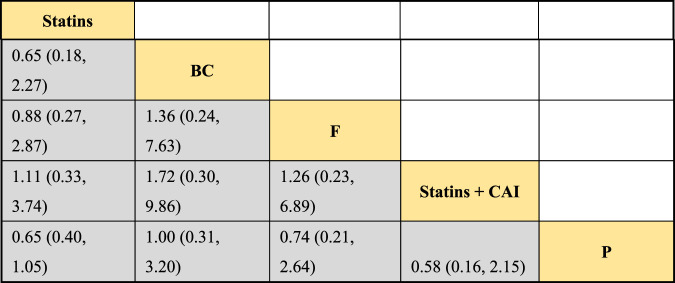
NMA results of the impact of DTRs on the change in LDL-C levels in PKT patients. (Statins denote Statin Medications; BC stands for Bile Acid Sequestrants; F refers to Fibrates; Statins + CAI means Statins Combined with Ezetimibe; and P represents Placebo).

**FIGURE 7:**
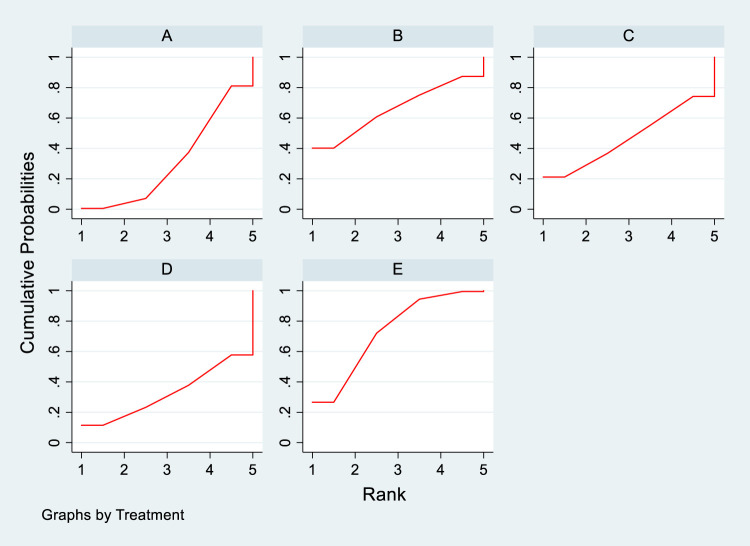
SUCRA plot of the change in LDL-C levels in PKT patients treated with DTRs [**(A)**: Statins, **(B)** BC, **(C)** F, **(D)** Statins + CAI, **(E)** P; Statins denote Statin Medications; BC stands for Bile Acid Sequestrants; F refers to Fibrates; Statins + CAI means Statins Combined with Ezetimibe; and P represents Placebo].

The included literature consisted of 9 studies analyzing the influence of DTRs on LDL-C levels in PKT patients, comprising a total of 4,865 patients across 5 intervention measures, including statins, fibrates, fish oil, statins combined with ezetimibe, and placebo (Supplementary Figure S4; Figures 5–7). NMA of LDL-C change magnitude revealed that the SUCRA values, ranked from highest to lowest, were as follows: statins (68.2%), statins combined with ezetimibe (67.5%), fish oil (53.4%), fibrates (34.5%), and placebo (26.5%).

#### 3.4.3 NMA results of the impact of DTRs on the magnitude of TC change in PKT patients

**FIGURE 8:**
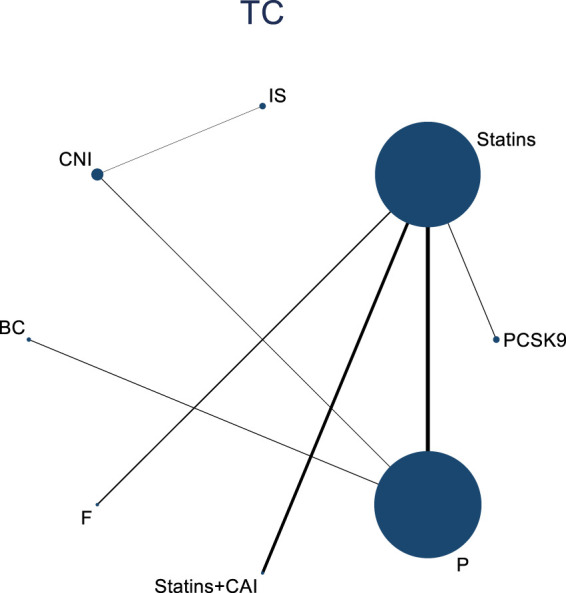
Network evidence relationship diagram of the effect of DTRs on the change in TC levels in PKT patients. (PCSK9 stands for Proprotein Convertase Subtilisin/Kexin Type 9 Inhibitors; Statins refer to Statin Medications; IS denotes Immunosuppressants; CNI stands for Calcineurin Inhibitors; BC refers to Bile Acid Sequestrants; F represents Fibrates; Statins + CAI means Statins Combined with Ezetimibe; Steroids refer to Corticosteroids; and P stands for Placebo.)

**FIGURE 9:**
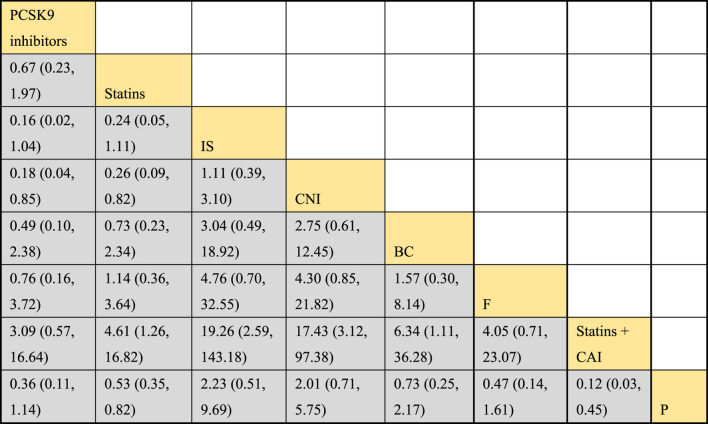
NMA results of the effect of DTRs on the change in TC levels in PKT patients. (PCSK9 stands for Proprotein Convertase Subtilisin/Kexin Type 9 Inhibitors; Statins refer to Statin Medications; IS denotes Immunosuppressants; CNI stands for Calcineurin Inhibitors; BC refers to Bile Acid Sequestrants; F represents Fibrates; Statins + CAI means Statins Combined with Ezetimibe; and P stands for Placebo.)

**FIGURE 10:**
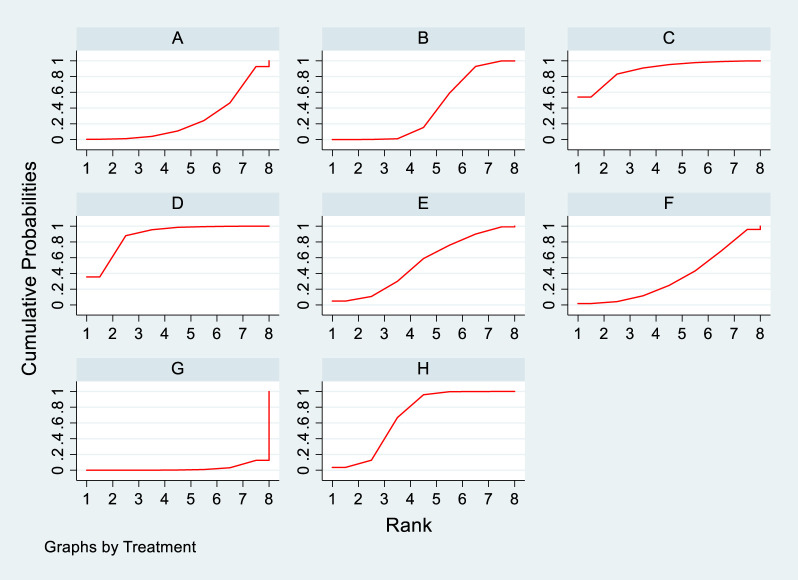
SUCRA diagram depicting the magnitude of TC changes in PKT patients treated with different therapeutic regimens [**(A)** for PCSK9 inhibitors, **(B)** for Statins, **(C)** for IS, **(D)** for CNI, **(E)** for BC, **(F)** for F, **(G)** for Statins + CAI, **(H)** for P; PCSK9 stands for Proprotein Convertase Subtilisin/Kexin Type 9 Inhibitors; Statins refer to Statin Medications; IS denotes Immunosuppressants; CNI stands for Calcineurin Inhibitors; BC refers to Bile Acid Sequestrants; F represents Fibrates; Statins + CAI means Statins Combined with Ezetimibe; and P stands for Placebo].

Twelve studies investigated the effects of DTRs on TC in PKT patients, with a total of 5,521 patients and 8 intervention measures, including PCSK9 inhibitors, statins, immunosuppressants, calcium channel blockers, bile acid sequestrants, fish oil, statins plus ezetimibe, and placebo. A NMA of TC change magnitude revealed that the SUCRA values, indicating the ranking of treatment efficacy, were highest for statins plus ezetimibe (97.6%), followed by PCSK9 inhibitors (74.3%), fish oil (64.3%), statins (61.6%), bile acid sequestrants (47.2%), placebo (31.6%), calcium channel blockers (11.9%), and immunosuppressants (11.4%) (Supplementary Figure S5; Figures 8–10).

#### 3.4.4 Meta-analysis results of the impacts of DTRs on PKT patients’ TG change magnitude

**FIGURE 11:**
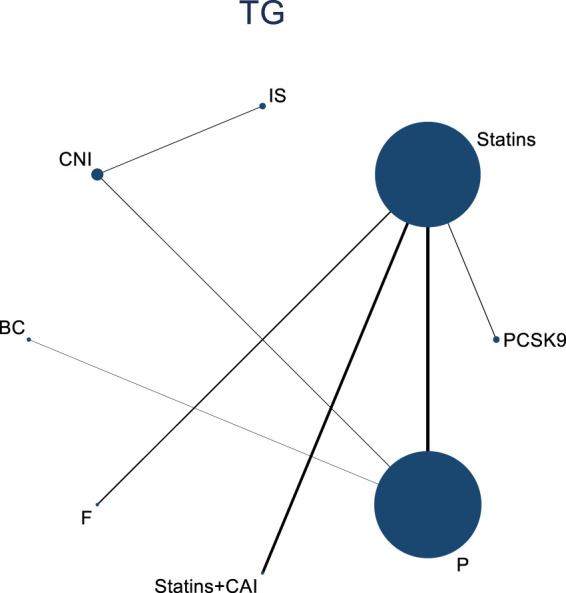
Network evidence relationship diagram of the effect of DTRs on the magnitude of TG changes in PKT patients. (PCSK9 stands for Proprotein Convertase Subtilisin/Kexin Type 9 Inhibitors; Statins refer to Statin Medications; IS denotes Immunosuppressants; CNI stands for Calcineurin Inhibitors; BC refers to Bile Acid Sequestrants; F represents Fibrates; Statins + CAI means Statins Combined with Ezetimibe; and P stands for Placebo).

**FIGURE 12:**
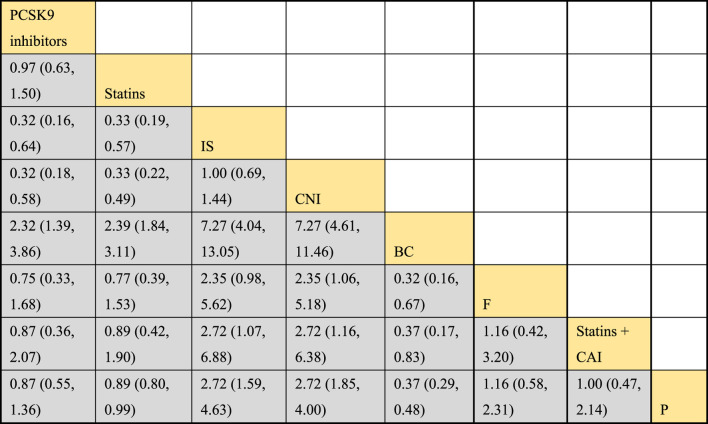
NMA results of the effect of DTRs on the magnitude of TG changes in PKT patients. (PCSK9 stands for Proprotein Convertase Subtilisin/Kexin Type 9 Inhibitors; Statins refer to Statin Medications; IS denotes Immunosuppressants; CNI stands for Calcineurin Inhibitors; BC refers to Bile Acid Sequestrants; F represents Fibrates; Statins + CAI means Statins Combined with Ezetimibe; and P stands for Placebo).

**FIGURE 13:**
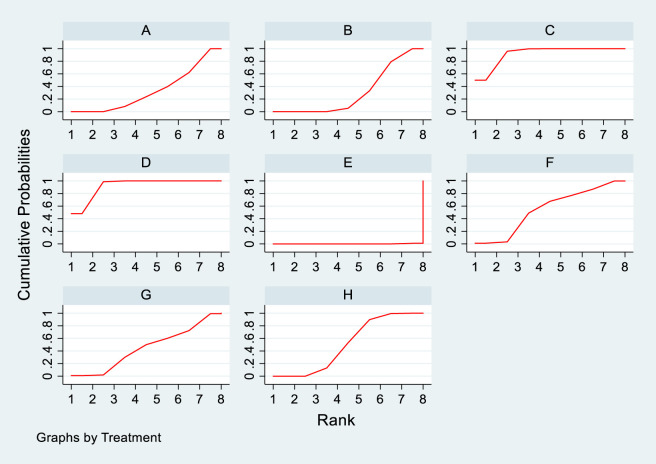
SUCRA plot of the changes in TG levels in PKT patients treated with DTRs [**(A)** for PCSK9 inhibitors, **(B)** for Statins, **(C)** for IS, **(D)** for CNI, **(E)** for BC, **(F)** for F, **(G)** for Statins + CAI, **(H)** for P; PCSK9 stands for Proprotein Convertase Subtilisin/Kexin Type 9 Inhibitors; Statins refer to Statin Medications; IS denotes Immunosuppressants; CNI stands for Calcineurin Inhibitors; BC refers to Bile Acid Sequestrants; **(F)** represents Fibrates; Statins + CAI means Statins Combined with Ezetimibe; and P stands for Placebo].

Twelve studies investigated the effects of DTRs on PKT patients’ TG levels, comprising a total of 5,521 patients and involving 8 intervention measures, including PCSK9 inhibitors, statins, immunosuppressants, calcium channel blockers, fibrates, fish oil, statins plus ezetimibe, and placebo (Supplementary Figure S6; Figures 11–13). NMA of TG change magnitude revealed that the SUCRA rankings, from highest to lowest, for PKT patients receiving DTRs were as follows: fibrates (99.9%), statins (68.9%), PCSK9 inhibitors (66.6%), statins plus ezetimibe (55.1%), placebo (49.2%), fish oil (45.0%), immunosuppressants (7.8%), and calcium channel blockers (7.6%).

#### 3.4.5 Meta-analysis results of the influence of DTRs on cardiovascular adverse events in PKT patients

**FIGURE 14:**
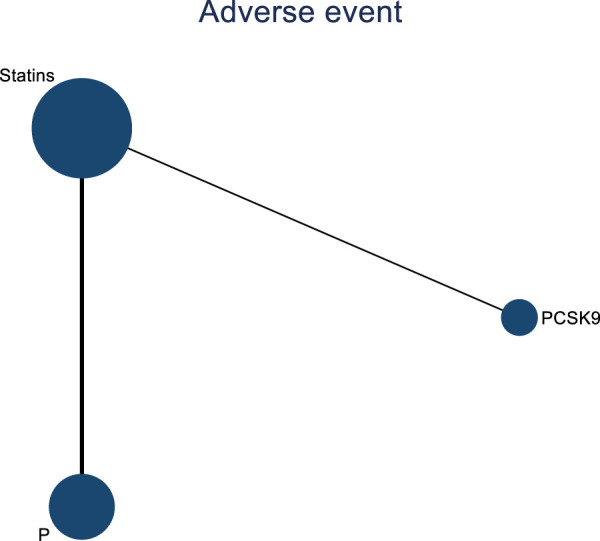
Network evidence map of the effects of DTRs on cardiovascular and cerebrovascular adverse events in PKT patients. (PCSK9 stands for Proprotein Convertase Subtilisin/Kexin Type 9 Inhibitors; Statins refer to Statin Medications; and P stands for Placebo).

Two studies assessed the impact of DTRs on cardiovascular adverse events in PKT patients, comprising a total of 561 patients and involving three intervention strategies: PCSK9 inhibitors, statins, and placebo (Figure 14).

#### 3.4.6 NMA results of the impact of treatment regimens on PKT patients’ graft failure

**FIGURE 15:**
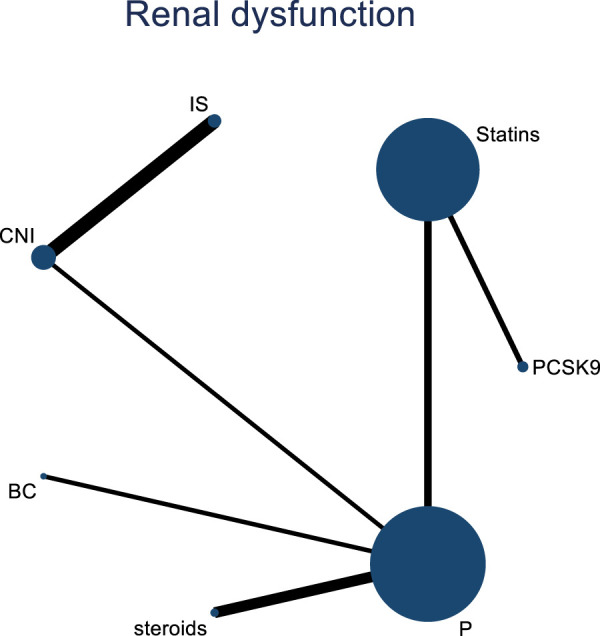
Network evidence map of the effects of DTRs on graft failure in PKT patients. (PCSK9 stands for Proprotein Convertase Subtilisin/Kexin Type 9 Inhibitors; Statins refer to Statin Medications; IS denotes Immunosuppressants; CNI stands for Calcineurin Inhibitors; BC refers to Bile Acid Sequestrants; Steroids represents Steroids; and P stands for Placebo).

**FIGURE 16:**
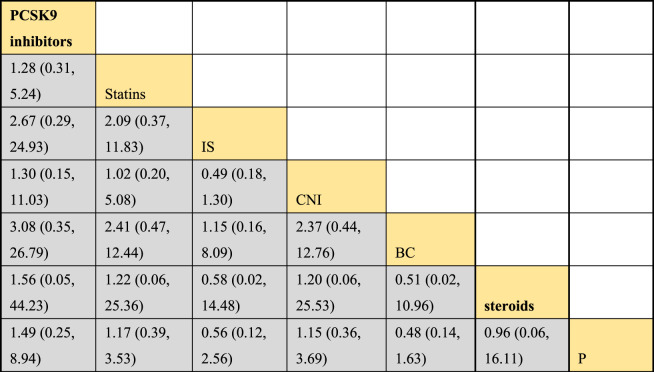
NMA results of the effects of DTRs on graft failure in PKT patients. (PCSK9 stands for Proprotein Convertase Subtilisin/Kexin Type 9 Inhibitors; Statins refer to Statin Medications; IS denotes Immunosuppressants; CNI stands for Calcineurin Inhibitors; BC refers to Bile Acid Sequestrants; Steroids represents Steroids; and P stands for Placebo).

**FIGURE 17:**
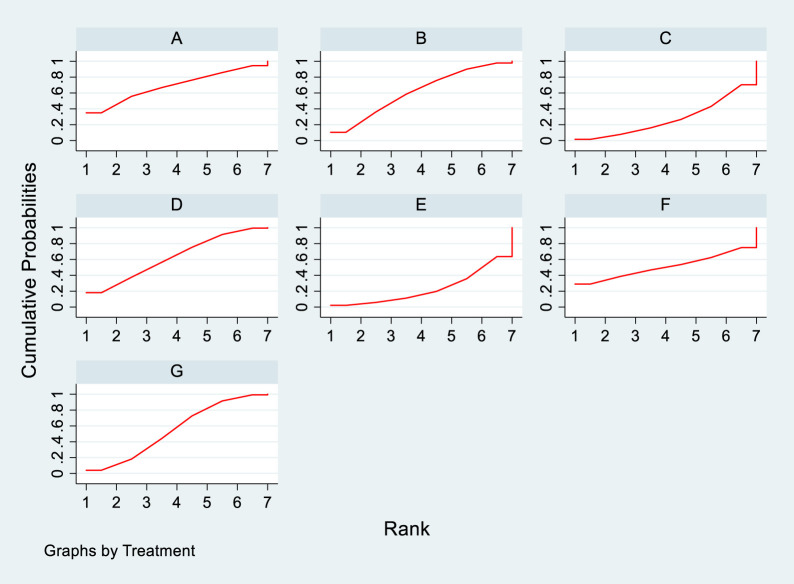
SUCRA plot of the impact of DTRs on PKT patients’ graft failure. [**(A)** for PCSK9 inhibitors, **(B)** for Statins, **(C)** for IS, **(D)** for CNI, **(E)** for BC, **(F)** for Steroids, **(G)** for P; PCSK9 stands for Proprotein Convertase Subtilisin/Kexin Type 9 Inhibitors; Statins refer to Statin Medications; IS denotes Immunosuppressants; CNI stands for Calcineurin Inhibitors; BC refers to Bile Acid Sequestrants; Steroids represents Steroids; and P stands for Placebo].

Incorporating 8 studies, the analysis examined the impact of treatment regimens on PKT patients’ graft failure, encompassing a total of 3,114 patients and 7 intervention approaches, including PCSK9 inhibitors, statins, immunosuppressants, calcium channel blockers, fibrates, corticosteroids, and placebos (Supplementary Figure S7; Figures 15–17). Conducting a NMA on graft failure, it was observed that the SUCRA values, indicating the likelihood of reducing graft failure occurrence, ranked in descending order as follows for the various treatment approaches: PCSK9 inhibitors (69.0%), calcium channel blockers (63.0%), statins (61.5%), placebo (55.1%), corticosteroids (51.8%), immunosuppressants (27.1%), and fibrates (22.5%).

#### 3.4.7 NMA results regarding the impact of DTRs on acute rejection reactions in PKT patients

**FIGURE 18:**
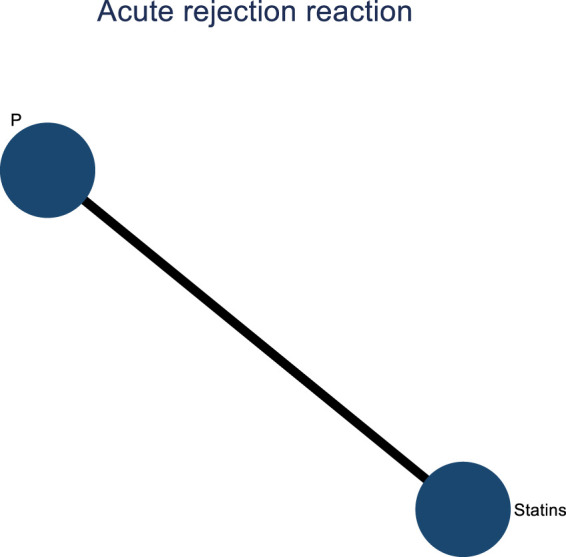
Network evidence diagram of the impact of DTRs on acute rejection reactions in PKT patients. (Statins and P stand for Statin Medications and Placebo, respectively).

Two studies examined the impact of DTRs on acute rejection reactions in PKT patients. These studies comprised a total of 2,176 patients and investigated two intervention measures, including statins and a placebo (Figure 18).

#### 3.4.8 NMA results on the impact of DTRs on all-cause mortality in PKT patients

**FIGURE 19:**
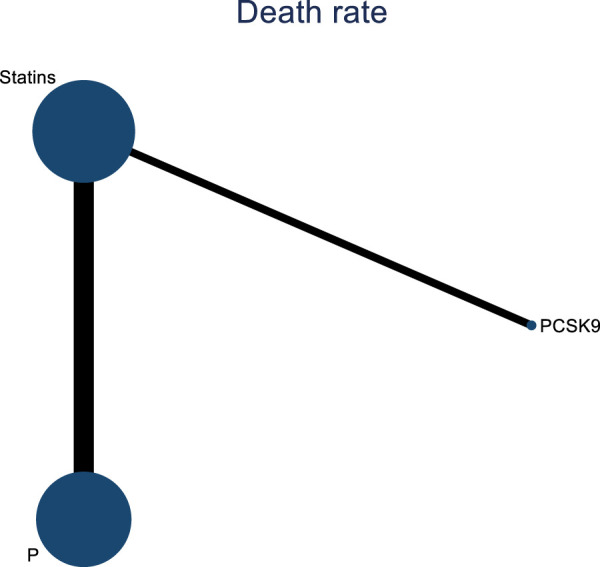
Network evidence diagram of the impact of DTRs on all-cause mortality in PKT patients. (PCSK9 stands for Proprotein Convertase Subtilisin/Kexin Type 9 Inhibitors; Statins denote Statin Medications; and P refers to Placebo).

**FIGURE 20:**
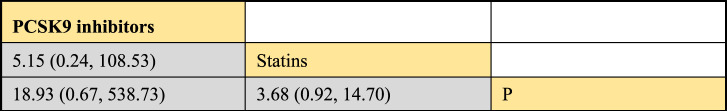
NMA results of the impact of DTRs on all-cause mortality in PKT patients. (PCSK9 stands for Proprotein Convertase Subtilisin/Kexin Type 9 Inhibitors; Statins denote Statin Medications; and P refers to Placebo).

**FIGURE 21:**
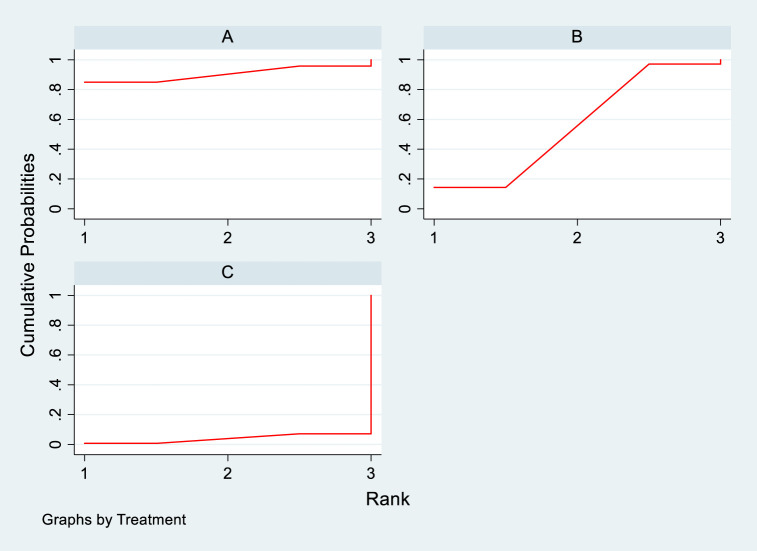
SUCRA plot of all-cause mortality in PKT patients treated with various treatment strategies [**(A)** for PCSK9 inhibitors, **(B)** for statins, **(C)** for P; PCSK9 stands for Proprotein Convertase Subtilisin/Kexin Type 9 Inhibitors; Statins denote Statin Medications; and P refers to Placebo].

Four studies analyzed the impact of DTRs on all-cause mortality in PKT patients, comprising a total of 2,699 patients and involving three intervention measures, including PCSK9 inhibitors, statins, and placebo (Supplementary Figure S8; Figures 19–21). A NMA on all-cause mortality revealed that the SUCRA values, indicating the effectiveness in reducing all-cause mortality, ranked from highest to lowest as follows: PCSK9 inhibitors (90.5%), statins (55.8%), and placebo (3.7%).

### 3.5 Bias analysis

**FIGURE 22:**
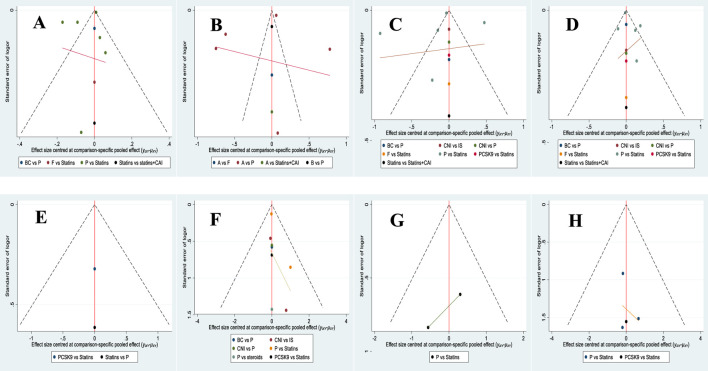
Comparison of publication bias in the effects of various treatment strategies on lipid reduction and safety in PKT patients-corrected funnel plot. [**(A)** for HDL-C, **(B)** for LDL-C, **(C)** for TC, **(D)** for TG, **(E)** for cardiovascular adverse events, **(F)** for transplant failure, **(G)** for acute rejection, **(H)** for all-cause mortality].

By plotting a standard funnel plot for the analysis of publication bias in the included literature, it was observed that the funnel plot exhibited good symmetry, and the included studies were evenly distributed, indicating a minimal publication bias in the study designs of the included literature. The standard funnel plot is presented in Figure 22.

### 3.6 GRADE evidence quality assessment

This study included a total of eight outcome measures, with HDL-C, LDL-C, TC, TG, and renal transplant failure as intermediate quality evidence, and cardiovascular and cerebrovascular adverse reactions, acute rejection, and all-cause mortality as low-quality evidence (Table 2).

**TABLE 2:** Results of GRADE evidence quality assessment included in the study.

Final result	Number of documents	Sample size	Risk of bias	Inconsistency	Indirectness	Imprecision	Publication bias	Evidence quality
HDL-C	9	4,865	1^a^	0	0	0	0	Medium level
LDL-C	9	4,865	0	0	0	1^b^	0	Medium level
TC	12	5,521	1^a^	0	0	0	0	Medium level
TG	12	5,521	1^a^	0	0	0	0	Medium level
Adverse cardiovascular and cerebrovascular reactions	2	561	1^a^	0	0	1^b^	0	Lower level
Renal transplant failure	8	3,114	1^a^	0	0	0	0	Medium level
Acute rejection reaction	2	2,176	1^a^	0	0	1^b^	0	Lower level
All-cause mortality rate	4	2,699	1^a^	0	0	1^b^	0	Lower level

^a^ The description of randomized grouping, allocation concealment, and blinding in the included studies was unclear.

^b^ The sample size included in the study was small, the confidence interval was wide, and it crossed the invalid line.

## 4 Discussion

PKT patients often face the necessity of using immunosuppressants, which can affect metabolism and lead to dyslipidemia (Fuentes-Orozco et al., 2018; Jafari et al., 2017). Long-term administration of these immunosuppressants is typically required to prevent organ rejection. Different classes of immunosuppressants may have varying impacts on lipid metabolism, resulting in dyslipidemia. For instance, CNIs such as cyclosporine and tacrolimus have been shown to influence lipid metabolism. Cyclosporine can elevate levels of LDL-C and TC, while tacrolimus may increase TG levels. Additionally, mycophenolate mofetil (MMF) generally does not significantly affect lipid levels, but its combination with CNIs may exacerbate lipid abnormalities. Corticosteroids may also contribute to dyslipidemia, particularly with long-term use (Lepre et al., 1999; Pipeleers et al., 2021). Pharmacological treatment remains a crucial strategy for managing dyslipidemia, and selecting medications with high efficacy and safety profiles is essential for promoting patient health.

To further evaluate the efficacy of lipid-lowering therapies in managing elevated lipid levels in PKT patients, this study conducted a systematic review and network meta-analysis of lipid management medications for these patients. The selection of medications for network meta-analysis was based on their ability to adjust lipid levels in PKT patients, as well as their safety and tolerability. For instance, statins are widely used due to their potent LDL-C lowering effects; ezetimibe, a cholesterol absorption inhibitor, can enhance the efficacy of statins; fibrates are primarily used to reduce triglyceride levels; and PCSK9 inhibitors have garnered attention for their significant LDL-C lowering effects and favorable safety profile. Our study results indicated that, in the NMA of HDL-C change magnitude, the SUCRA values for PKT patients treated with different therapeutic regimens ranked from highest to lowest as follows: statins + ezetimibe (70%), placebo (61.5%), fibrates (57.2%), statins (44.1%), and fish oil (17.3%). In the NMA of LDL-C change magnitude, the SUCRA values for PKT patients treated with different therapeutic regimens ranked from highest to lowest as follows: statins (68.2%), statins + ezetimibe (67.5%), fish oil (53.4%), fibrates (34.5%), and placebo (26.5%). In the NMA of TC change magnitude, the SUCRA values for PKT patients treated with different therapeutic regimens ranked from highest to lowest as follows: statins + ezetimibe (97.6%), PCSK9 inhibitors (74.3%), fish oil (64.3%), statins (61.6%), fibrates (47.2%), placebo (31.6%), calcium channel blockers (11.9%), and immunosuppressants (11.4%). In the NMA of TG change magnitude, the SUCRA values for PKT patients treated with different therapeutic regimens ranked from highest to lowest as follows: fibrates (99.9%), statins (68.9%), PCSK9 inhibitors (66.6%), statins + ezetimibe (55.1%), placebo (49.2%), fish oil (45.0%), immunosuppressants (7.8%), and calcium channel blockers (7.6%). In PKT patients, common lipid abnormalities include hypercholesterolemia, hypertriglyceridemia, and low levels of HDL-C. These abnormalities are closely associated with an increased risk of CVD, which is a leading cause of non-infectious mortality in PKT patients. Low HDL-C levels are generally linked to the progression of atherosclerosis, whereas elevated HDL-C levels are considered to have an atheroprotective effect. Similarly, increased levels of LDL-C and TG can accelerate atherosclerosis, thereby increasing the risk of coronary artery disease and other cardiovascular events. In terms of reducing lipid levels in PKT patients, the combination of statins and ezetimibe offers a significant advantage. Statins primarily function by inhibiting cholesterol synthesis, which can significantly increase HDL-C levels. Ezetimibe, a cholesterol absorption inhibitor, can also elevate HDL-C levels. The combination of statins and ezetimibe can have a synergistic effect, substantially increasing HDL-C levels and thus promoting cholesterol reverse transport and metabolism, which reduces the risk of atherosclerosis (Kosch et al., 2004). Research indicated that ezetimibe can significantly lower TG levels by inhibiting their absorption in the intestine [(Lebranchu et al., 2009); (Lal et al., 1995)]. In PKT patients, lipid metabolism abnormalities are often exacerbated by chronic renal insufficiency, and ezetimibe can effectively address these issues. PKT patients frequently exhibit multiple types of dyslipidemia, including elevated LDL-C, high TG, and low HDL-C. The combination of statins and ezetimibe can address multiple lipid abnormalities simultaneously, resulting in more comprehensive lipid regulation (Krämer et al., 2016). Long-term, effective lipid management is crucial for reducing the incidence of cardiovascular events, improving patient quality of life, and prolonging the survival of transplanted organs. By lowering LDL-C levels, the progression of atherosclerosis can be mitigated, thereby reducing the risk of myocardial infarction and stroke. Additionally, the use of medications with fewer side effects can diminish patient resistance to treatment and improve adherence. Cost-effectiveness analyses indicate that although some newer lipid-lowering medications may be expensive, their significant reduction in cardiovascular event risk may make them a cost-effective option in the long run.

PKT patients with dyslipidemia are at increased risk of cardiovascular and cerebrovascular adverse events. Prolonged dyslipidemia, especially high cholesterol and high TGs, increases the risk of atherosclerosis, leading to vascular narrowing, plaque formation, and even thrombosis, thereby elevating the risk of cardiovascular events such as angina, myocardial infarction, and stroke. Moreover, dyslipidemia adversely affects vascular endothelial cells and smooth muscle cells, resulting in vascular dysfunction, thrombosis, and vascular injury (Flechner et al., 2002). Particularly after kidney transplantation, the use of immunosuppressive drugs may exacerbate dyslipidemia, further increasing the risk of cardiovascular adverse events. Additionally, high cholesterol and other lipid abnormalities may affect the function of the immune system, particularly under immunosuppressive therapy, potentially exacerbating immune dysfunction and increasing the risk of acute rejection (Fassett et al., 2010). Lipid abnormalities also impact vascular health, increasing the risk of endothelial damage, plaque formation, and other adverse effects, which may negatively affect the perfusion and function of the transplanted kidney, thereby increasing the risk of acute rejection. Studies have shown that PKT patients with lipid abnormalities may have an increased risk of acute rejection and mortality compared to those with normal lipid levels (Jardine et al., 2005). Therefore, analyzing the role of lipid-lowering drugs in reducing the incidence of cardiovascular adverse events is of paramount importance.

To further investigate the impact of lipid-lowering drugs on cardiovascular adverse events and all-cause mortality, this study conducted a meta-analysis. The results showed that in the analysis of PKT failure, the SUCRA for reducing the incidence of transplant failure in PKT patients receiving DTRs was highest for PCSK9 inhibitors (69.0%), followed by calcium channel blockers (63.0%), statins (61.5%), placebo (55.1%), steroids (51.8%), immunosuppressants (27.1%), and fibrates (22.5%). In the analysis of all-cause mortality, the SUCRA for reducing all-cause mortality in PKT patients receiving DTRs was highest for PCSK9 inhibitors (90.5%), followed by statins (55.8%) and placebo (3.7%). It was observed that PCSK9 inhibitors demonstrate significant advantages in reducing adverse events and mortality rates in PKT patients. PCSK9 primarily functions to facilitate the degradation of low-density lipoprotein receptors (LDL receptors), leading to an increase in LDL-C in the bloodstream (Pascual et al., 2005b). PCSK9 inhibitors work by blocking the binding of PCSK9 to LDL receptors, thereby increasing the quantity of LDL receptors in the liver, promoting the clearance of LDL-C, and consequently reducing LDL-C levels in the bloodstream (Artz et al., 2003; Santos et al., 2001). In PKT patients, PCSK9 inhibitors can significantly lower LDL-C, thereby reducing the risk of cardiovascular events. PKT patients often face increased cardiovascular risks, so lowering LDL-C levels can effectively prevent asthma attacks (Norby et al., 2009). PCSK9 inhibitors may reduce the formation of atherosclerotic plaques, potentially contributing to a reduction in immune system activation and inflammatory responses, thereby lowering the risk of acute rejection (Melchor and Gracida, 1998). PCSK9 inhibitors help improve endothelial function, reduce vascular damage and thrombus formation, thereby protecting the vascular health of the transplanted kidney, reducing damage to transplant kidney function, and possessing positive clinical implications (Kliem et al., 1996).

This study primarily relied on existing literature, which may be subject to publication bias, with positive results being more likely to be published. Furthermore, the included studies may vary in design, sample size, and patient characteristics, potentially introducing heterogeneity. Notably, differences between control and intervention groups across studies may affect the evaluation of the efficacy of different lipid-lowering drugs. For instance, some studies may have used varying control drugs or placebos, while others might have employed different doses of the same drug. These discrepancies could lead to inconsistencies in results, thereby affecting our understanding of the relative efficacy of various treatment regimens. Additionally, inconsistencies in drug dosages and treatment durations across studies may also impact the consistency of efficacy assessments. Lastly, this study did not sufficiently account for the effects of factors such as race and gender on drug efficacy, which represents a limitation. Future research should further investigate the underlying mechanisms of lipid abnormalities, particularly those associated with immunosuppressants. For example, immunosuppressants such as cyclosporine and tacrolimus have been shown to affect lipid metabolism. Moreover, additional randomized controlled trials are needed to assess the long-term effects of different drug combinations, while considering a broader range of demographic variables, including age, sex, race, and comorbidities, to better understand how these factors influence treatment outcomes. Additionally, exploring new biomarkers and personalized treatment approaches could enhance the precision of lipid management strategies.

To enhance the comparability of future meta-analyses, more standardized clinical trials are needed in this field. These trials should adhere to uniform design principles, including well-defined control group settings, consistent treatment doses and durations, and comprehensive documentation of patient demographic characteristics and other relevant variables. Such standardization will enable future research to provide more reliable data, thereby better guiding clinical practice and policy development.

## 5 Conclusion

The combination of statins and ezetimibe can better reduce lipid levels in PKT patients, while PCSK9 inhibitors can lower the incidence of adverse reactions and all-cause mortality in these patients, demonstrating high efficacy and safety. Therefore, they have the potential for widespread clinical application.

## Data Availability

The original contributions presented in the study are included in the article/Supplementary Material, further inquiries can be directed to the corresponding author.
