# Supplementary material for: Management of blood lipids in post-kidney transplant patients: a systematic review and network meta-analysis
Source: Front Pharmacol. 2024 Oct 8;15:1440875. doi: 10.3389/fphar.2024.1440875 (PMC11493609; doi:10.3389/fphar.2024.1440875)
Supplement: Supplementary file 1 [file Presentation1.pdf]

Supplementary Figures

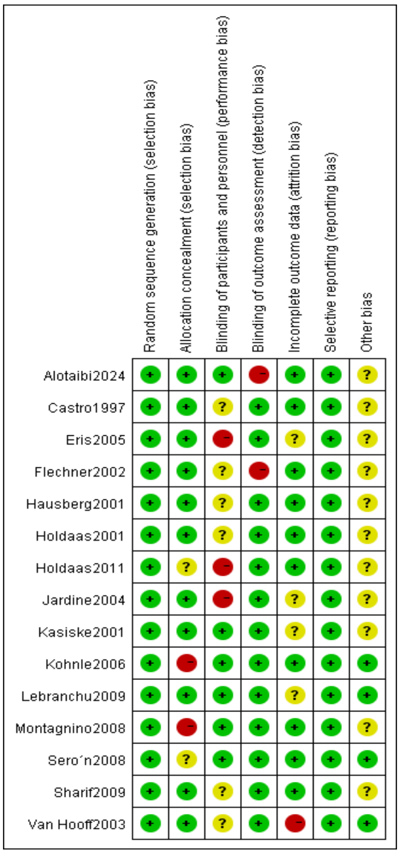

Supplementary Figure 1. Summary assessment of bias risk.

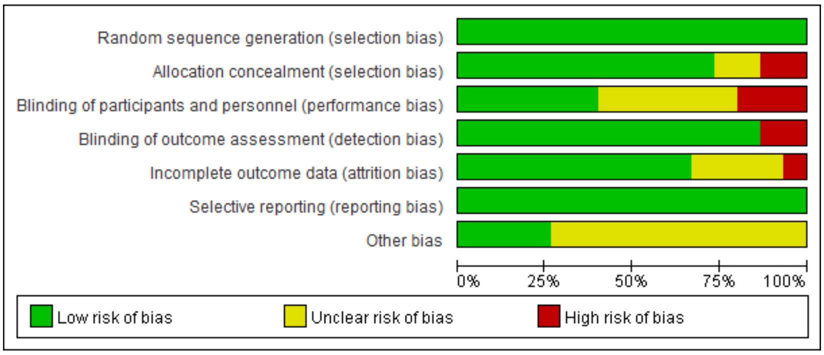

Supplementary Figure 2. Overall assessment of bias risk.

A

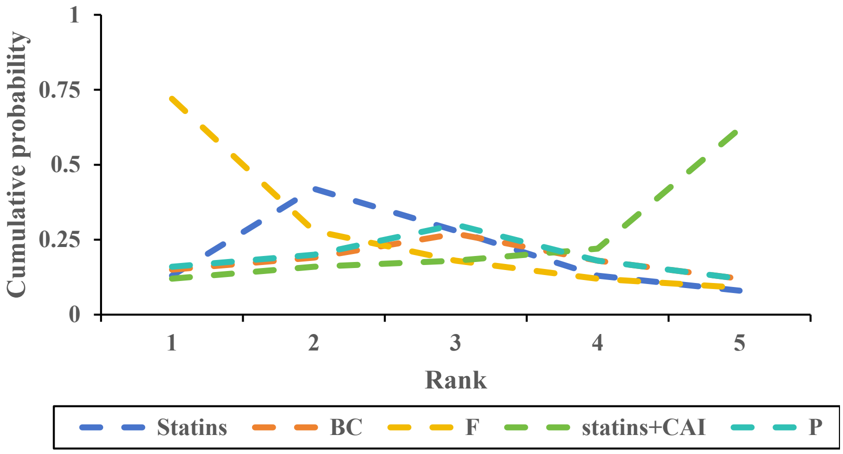

B

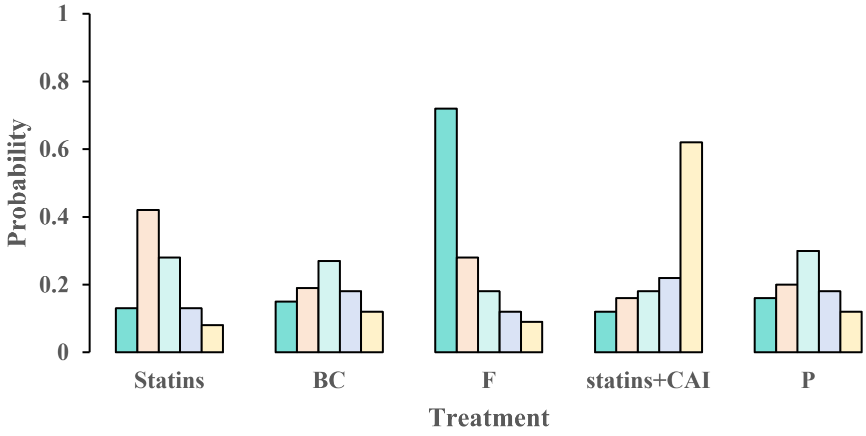

Supplementary Figure 3. Probability ranking chart and strip probability chart of HDL-C changes. (A is the probability ranking chart, and B is the striped probability chart; Statins denote Statin Medications; BC stands for Bile Acid Sequestrants; F refers to Fibrates; Statins+CAI means Statins Combined with Ezetimibe; and P represents Placebo)

A

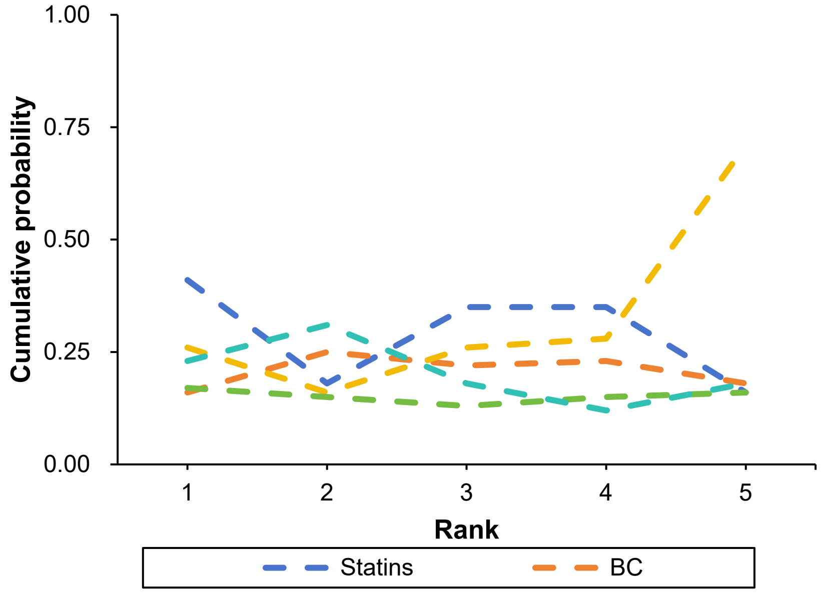

B

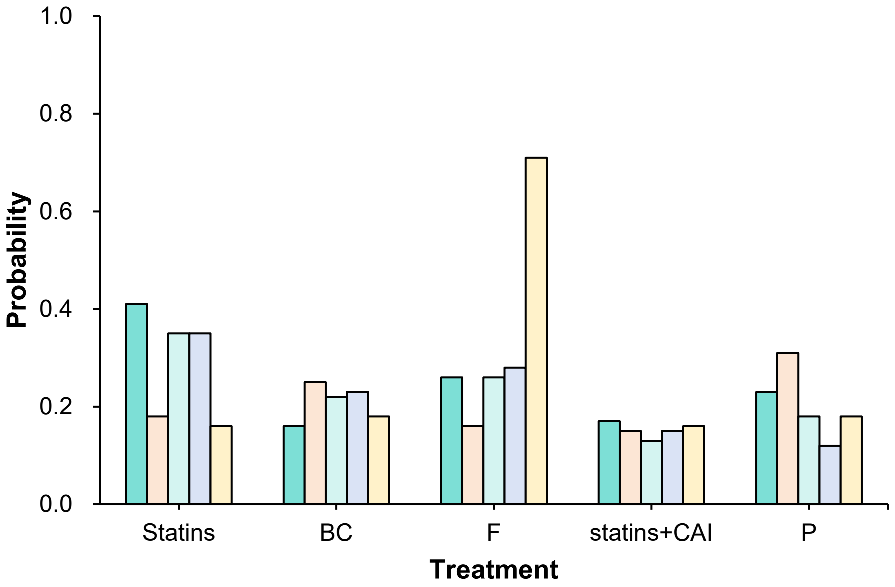

Supplementary Figure 4. Probability ranking chart and strip probability chart of LDL-C changes. (A is the probability ranking chart, and B is the striped probability chart; Statins denote Statin Medications; BC stands for Bile Acid Sequestrants; F refers to Fibrates; Statins+CAI means Statins Combined with Ezetimibe; and P represents Placebo)

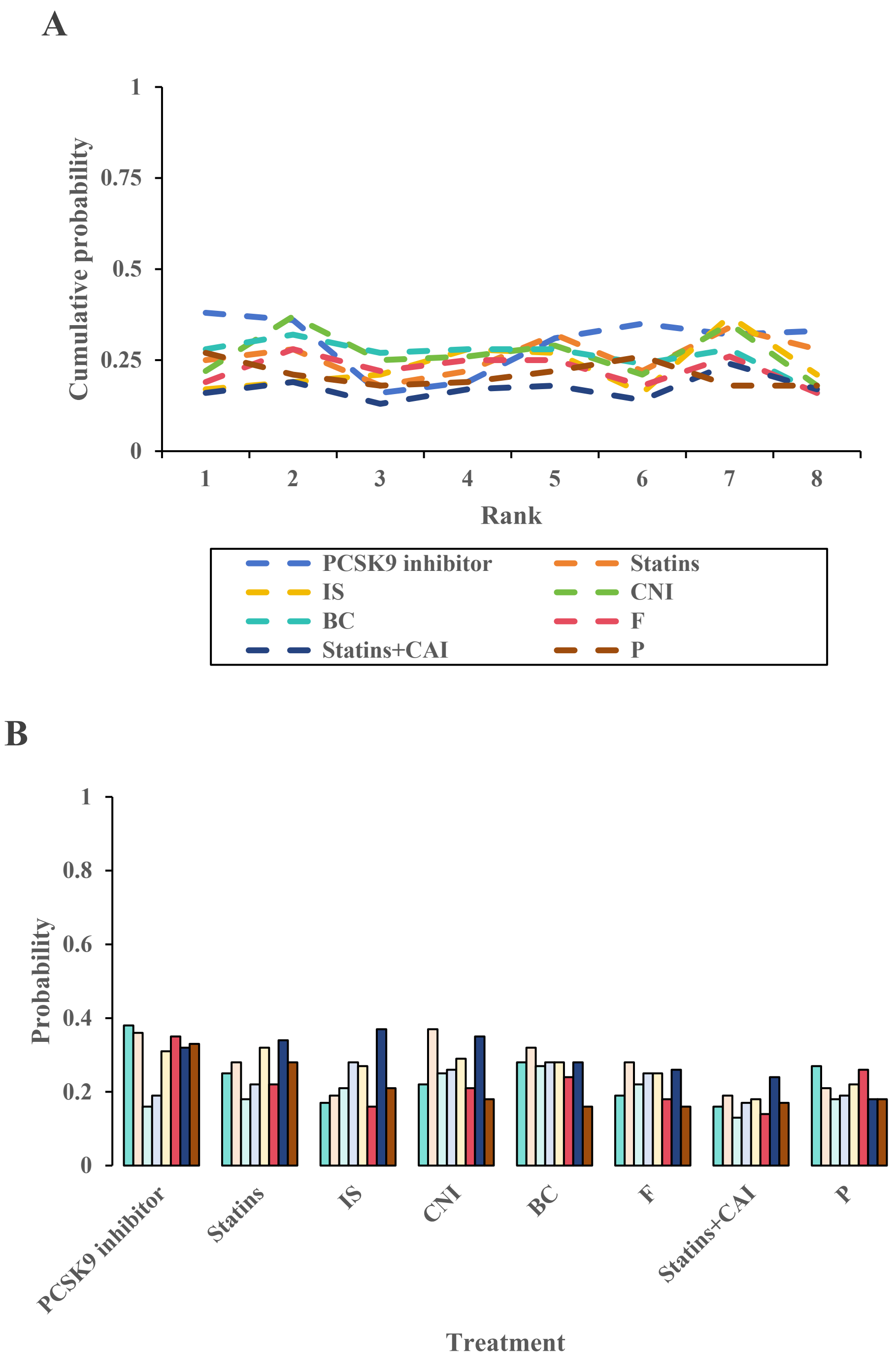

Supplementary Figure 5. Probability ranking chart and strip probability chart of TC changes. (A is the probability ranking chart, and B is the striped probability chart; PCSK9 stands for Proprotein Convertase Subtilisin/Kexin Type 9 Inhibitors; Statins refer to Statin Medications; IS denotes Immunosuppressants; CNI stands for Calcineurin Inhibitors; BC refers to Bile Acid Sequestrants; F represents Fibrates; Statins+CAI means Statins Combined with Ezetimibe; and P stands for Placebo)

A

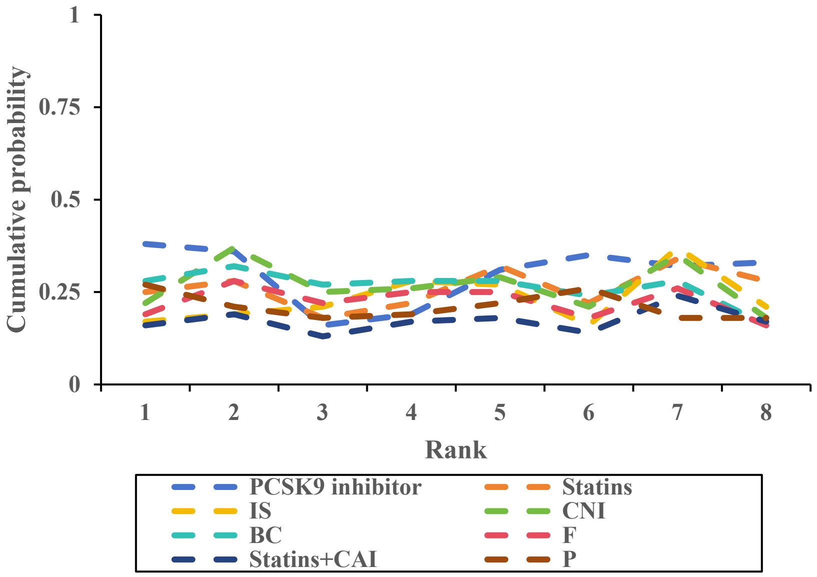

B

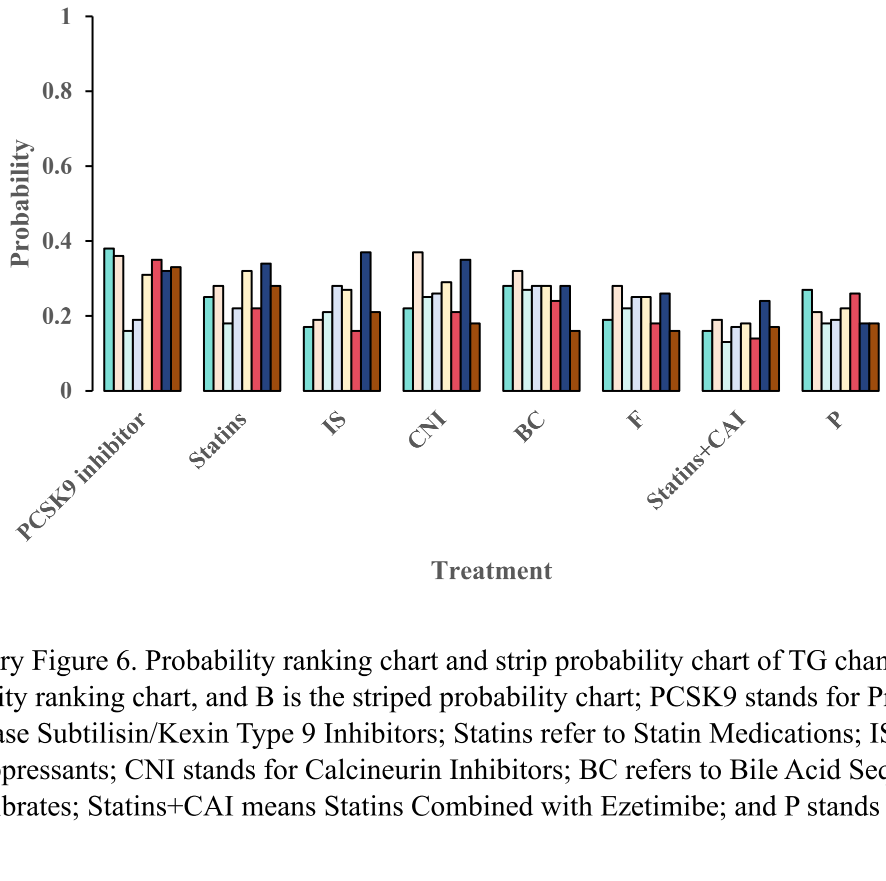

Supplementary Figure 6. Probability ranking chart and strip probability chart of TG changes. (A is the probability ranking chart, and B is the striped probability chart; PCSK9 stands for Proprotein Convertase Subtilisin/Kexin Type 9 Inhibitors; Statins refer to Statin Medications; IS denotes Immunosuppressants; CNI stands for Calcineurin Inhibitors; BC refers to Bile Acid Sequestrants; F represents Fibrates; Statins+CAI means Statins Combined with Ezetimibe; and P stands for Placebo.)

A

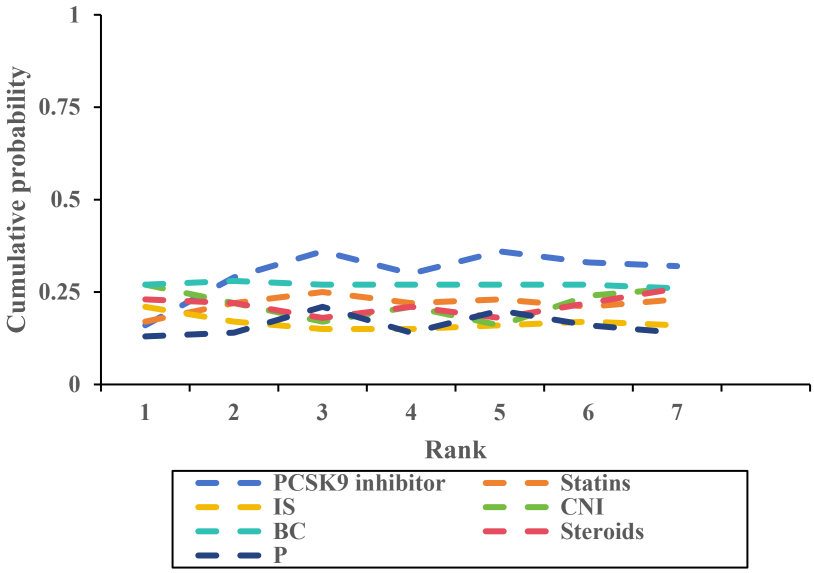

B

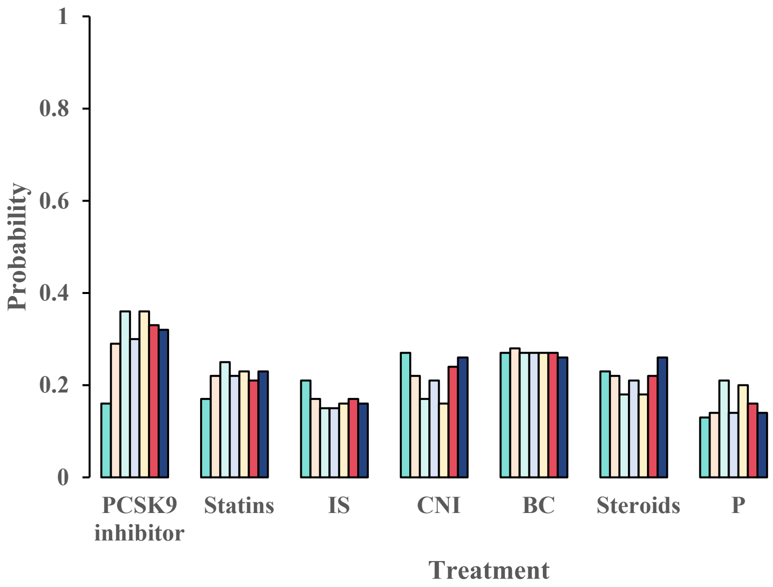

Supplementary Figure 7. Probability ranking chart and segmented probability chart of kidney transplant failure. (A is the probability ranking chart, and B is the striped probability chart; PCSK9 stands for Proprotein Convertase Subtilisin/Kexin Type 9 Inhibitors; Statins refer to Statin Medications; IS denotes Immunosuppressants; CNI stands for Calcineurin Inhibitors; BC refers to Bile Acid Sequestrants; Steroids represents Steroids; and P stands for Placebo.)

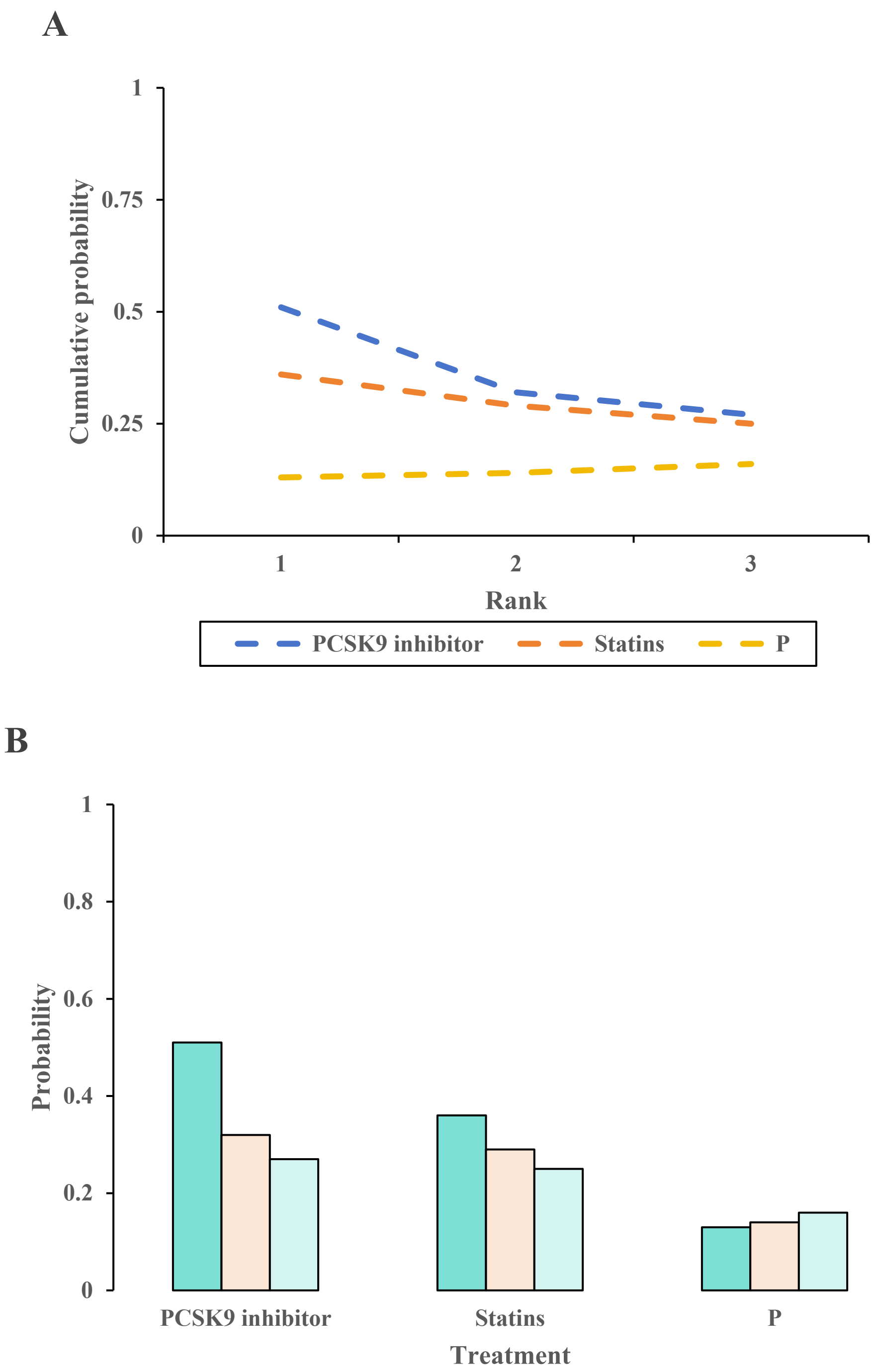

Supplementary Figure 8. Probability ranking chart and strip probability chart of all-cause mortality rate. (A is the probability ranking chart, and B is the striped probability chart; PCSK9 stands for Proprotein Convertase Subtilisin/Kexin Type 9 Inhibitors; Statins denote Statin Medications; and P refers to Placebo.)
